# Cell Therapy for Ischemic Stroke with Nanoparticle-Labeled 293T Cells and Bone Marrow-Derived Mesenchymal Stem Cells: A Feasibility Study

**DOI:** 10.3390/pharmaceutics18060704

**Published:** 2026-06-08

**Authors:** Kuo-Feng Huang, Te-Sun Chou, Jong-Kai Hsiao

**Affiliations:** 1 Division of Neurosurgery, Taipei Tzu Chi Hospital, Buddhist Tzu Chi Medical Foundation, New Taipei City 23142, Taiwan; kuofeng1234@gmail.com; 2 School of Medicine, Tzu Chi University, Hualien 97004, Taiwan; 3 Department of Medical Imaging, Taipei Tzu-Chi Hospital, Buddhist Tzu-Chi Medical Foundation, New Taipei City 23142, Taiwan

**Keywords:** ischemic stroke, mesenchymal stem cells, 293T cells, choroid plexus, microglia, magnetic resonance imaging

## Abstract

**Background/Objectives:** Stroke remains the second leading cause of death worldwide, and cell therapy is among the most actively investigated strategies for its treatment. Recent transcriptomic evidence has revealed that 293T cells—the most widely used transient transfection model—possess a neural crest/neuronal lineage, making them a candidate for acute neural tissue engineering. **Methods:** We implanted iron oxide nanoparticle-labeled 293T cells (293T-ION) into an ischemic rat brain and monitored them longitudinally by 7T MRI, using ION-labeled bone marrow-derived mesenchymal stem cells (rMSC-ION) as a direct comparison. Functional recovery was assessed via mNSS and corner test scores, and infarct size was quantified by MRI. **Results**: 293T-ION cells showed no migration throughout the 40-day observation period, and functional recovery plateaued early compared with the progressive improvement seen with rMSC-ION. 293T cell implantation provoked pronounced, localized CD68-positive microglial hyperactivation at both implantation and ischemic sites, without migration toward the choroid plexus (CP). In contrast, rMSC-ION actively migrated to the CP and drove superior neuroplasticity marker expression (Ki67, Nestin, NeuN). **Conclusions**: 293T cells produce transient localized microglial activation and limited brain plasticity, whereas rMSCs drive sustained neurorestoration. Synergistic co-administration of these cell types may represent a future therapeutic strategy bridging hyper-acute and chronic recovery phases.

## 1. Introduction

Cerebrovascular disease is the second leading cause of death worldwide and a primary catalyst for severe, long-term disability [1]. Various cell therapies have been aggressively investigated for the treatment of ischemic stroke to extend the narrow therapeutic window of current recanalization protocols [2]. The most frequent cell types that enter clinical trials are bone marrow-derived mesenchymal stem cells (rMSC), neural and hematopoietic stem cells [3,4,5,6]. However, harvesting and expanding autologous stem cells is time-consuming and can mean that treatment exceeds the hyper-acute therapeutic golden time. Therefore, there is an urgent need to investigate other instantly accessible, suitable cell types.

The neuronal lineage of HEK 293 cells is attracting increased attention in the context of neurobiology [7]. HEK293 cells are derived from human embryonic kidney cells after transformation by an adenovirus 5 DNA fragment [7,8]. However, their rich expression of B-adrenergic receptors and neurofilaments initially raised compelling questions about their relationship with neuronal lineage cells [7]. Moreover, advanced transcriptome profiling from exon arrays has definitively demonstrated that HEK293 cells are derived from the adrenal medulla and early neural crest rather than mature renal epithelium [9]. The 293T cell line is derived from HEK 293 expressing SV40 T antigens, granting unparalleled proliferative capacity [10,11]. Interestingly, 293T cells exhibit a stem cell-like phenotype when cultured in a 3D sphere [12]. The highly proliferative nature of 293T cells, combined with their newly recognized neuroendocrine origin, is what caught our attention for their potential application in acute neural tissue engineering. However, their direct effect on neuroregulation in vivo is still unknown. 293T cells are mostly used for transient gene expression, in which neuromodulators may be expressed and packaged into vectors or exosomes [13]. Whether there is an intrinsic neuroregulatory role of unmodified HEK293T cells after direct intraparenchymal implantation into the ischemic brain is not known.

To evaluate such therapies, non-invasive tracking is mandatory. Iron oxide nanoparticles (IONs) have been used extensively for trafficking stem cells in living animals [14]. The full differentiation capacity of rMSC after ION labeling has been definitively proven [15]. Moreover, IONs allow the migration of cells in living animals to be dynamically visualized by magnetic resonance (MR) imaging, and further evidenced by Prussian blue staining in post-mortem histology [16]. The ferrous component of the particle is safely metabolized in the liver for the reconstitution of hemoglobin [17]. Among IONs, Ferucarbotran (Resovist^®^) is one of the safest for cell labeling, and it has been clinically utilized for over two decades. The labeling method simply involves adding Ferucarbotran into the culture medium, allowing spontaneous endocytosis without any transfecting agents that might harm the cells [14]. Most importantly, in vitro studies showed that labeling rMSC with Ferucarbotran has no deleterious influence on their trans-differentiation into neuron-like cells [18]. Consequently, labeling cells with IONs is a safe, efficient, and highly translational method for studying cell therapy in neurological diseases.

The host immune response, specifically governed by microglia, plays a bipolar role in the regulation of ischemic stroke recovery [19]. The phenotypic polarization of microglia—ranging from the classically activated, pro-inflammatory M1 state to the alternatively activated, tissue-remodeling M2 state—has several crucial effects on spontaneous recovery after cerebral infarction, heavily dictating anatomical and functional outcomes [20]. This study systematically compared the therapeutic effect, migratory tropism, and microglial interaction of bone marrow-derived rMSC and 293T cells. The primary endpoint was to assess the neurorestorative efficacy of 293T cell therapy by monitoring the anatomical area of infarction with serial MR imaging, sensorimotor functional scores, and histological analysis of the local immune response. The secondary endpoint was to directly compare the 293T cell therapeutic dynamics against the established regenerative profile of mesenchymal stem cells.

## 2. Materials and Methods

### 2.1. ION Labeling

We cultured human 293T cells and primary rat bone marrow-derived mesenchymal stem cells (rMSC) in a 10 cm^2^ culture dish overnight with Dulbecco’s modified Eagle medium (DMEM) supplemented with 10% fetal bovine serum (FBS) and antibiotics. Then we added 100 μg Fe/mL Ferucarbotran into each dish and cultured for another 24 h to allow spontaneous endocytosis. The excess Ferucarbotran was washed off with phosphate-buffered saline (PBS) three times. We collected these ION labeled 293T (293T-ION) and rMSC (rMSC-ION) by standard enzymatic trypsinization. This Ferucarbotran labeling protocol follows the method established and validated in our group’s prior work, which confirmed efficient ION internalization, preserved rMSC immunophenotype (CD29+/CD90+/CD34−), and demonstrated no adverse effects on cell viability, ROS production, or mitochondrial membrane potential [21].

### 2.2. Ischemic Reperfusion Animal Model

All animal procedures and protocols were followed in strict accordance with the institutional animal care committee of Taipei Tzu Chi Hospital. These procedures complied with the Guide for the Care and Use of Laboratory Animals (IACUC approval number: 106-IACUC-025). We used 13 male Sprague-Dawley rats in this study (293T-ION = 4, rMSC-ION = 5, and naïve choroid plexus isolation = 4). To enable formal statistical comparison against vehicle and no-injection baselines, behavioral and imaging data from two additional cohorts generated by the same surgical team under identical Middle Cerebral Artery Occlusion model(MCAO)–reperfusion procedure and identical assessment protocols at the same institution were incorporated as historical control arms: a no-injection ischemic Control cohort (n = 10) from our previously published study [21] and a saline-injected vehicle Control cohort (n = 8) from our prior publication [16]. The integrated four-group analytical framework is novel to the present manuscript; these reused cohorts have not previously been analyzed in this comparison structure. The ischemic–reperfusion stroke rat model was created by an experienced operator. Male Sprague rats at the age of 6–8 weeks were shaved on the neck and sanitized with 70% alcohol. Under isoflurane gas anesthesia, their necks were incised, and their muscles were dissected to expose the bilateral common carotid arteries. These rats were then immobilized on a rigid stereotactic frame. We removed their orbital bone at the right retro-orbital region and identified the right middle cerebral artery (MCA). We ligated the right MCA with a 10-0 nylon micro-suture and clamped the bilateral common carotid arteries with atraumatic clips for 90 min. We then released the ligated bilateral common carotid arteries and the MCA to induce profound reperfusion injury.

Pre-specified inclusion criteria for the two cell-therapy intervention cohorts (rMSC-ION and 293T-ION) generated in this study required a Day-1 MRI-quantified infarct volume of ≥50 mm^3^ to ensure detectable baseline injury sufficient to assess therapeutic effect; this a priori threshold follows STAIR preclinical recommendations [22] and established MCAO methodology [23,24]. Six additional rMSC-ION animals (Day-1 volume 31 ± 27 mm^3^; range 14–86 mm^3^) fell below this threshold and were excluded to maintain comparable injury severity with the 293T-ION cohort (mean Day-1 volume 185 ± 146 mm^3^) and avoid a regression-to-the-mean confound; all included intervention animals had Day-1 infarct volume ≥63.6 mm^3^. The historical no-injection ischemic Control cohort [21] and saline-injected vehicle Control cohort [16] were used as published, without retroactive application of this threshold. The excluded animals remain unpublished and are available for a future severity-stratified analysis.

Immediately following reperfusion, we injected either 293T-ION (*n* = 4) or rMSC-ION (*n* = 5) at a highly concentrated cell number of one million in 10 μL sterile PBS directly into the right corpus callosum. The specific injection coordinates were adjusted as described previously: anterior–posterior, 0 mm; medial-lateral, 2.0 mm; and dorsal-ventral, 3.0 mm relative to bregma [16].

### 2.3. In Vitro MRI

We used a clinical grade 1.5-T MRI system (MAGNETOM Aera, Siemens Healthineers, Erlangen, Germany) for in vitro cellular MR imaging to confirm labeling efficacy. Cells at the number of one million with or without ION labeling were harvested, collected in a 200 μL tube, and placed in a homemade water tank phantom to eliminate susceptibility artifacts. The tank was positioned in an 8-channel head coil for imaging. Three-dimensional T2-weighted fast spin-echo pulse sequences were used (TR/TE = 1300/266 ms). The slice thickness was 1.0 mm, and the field of view was 14 cm × 9.8 cm in the transverse plane with two repetitions. The images were further processed using syngo fastView (version VX57M, Siemens Healthineers, Erlangen, Germany) and analyzed with ImageJ software (Version 1.54f, NIH, Bethesda, Rockville, MD, USA).

### 2.4. In Vivo MRI

We utilized a high-field 7T MRI system (Biospec 70/30; Bruker, Ettlingen, Germany) for longitudinal rat brain imaging. The subjects were anesthetized with isoflurane, pre-scanned for localization, and then T2-weighted images focusing on the brain with a scanning field of 3 × 3 cm^2^ were acquired at multiple post-implantation intervals (e.g., days 1, 8, 15, 22, 29, 36). The following MR parameters were optimized: repetition time/echo time = 5000/56 ms; resolution = 256 × 256 pixels; slice thickness = 5 mm. The temporal recovery of the stroke volume was mathematically measured as (100 –) × 100, which was described previously [16].

### 2.5. Disability Score Evaluation After Ischemic–Reperfusion Injury

We evaluated the physiological disability status of the ischemia–reperfusion injured rats weekly using the modified Neurological Severity Score (mNSS) and the corner test as described previously [16]. These rigorous tests assess the complex sensorimotor disabilities of neurologically wounded rats.

Briefly, in the mNSS, sensorimotor dysfunction is categorized as disabilities in walking, righting, balancing, placing, and body resistance. Disabilities in each item are scored by adding one point. Up to 18 points is regarded as a maximal, catastrophic disability.

The corner test assesses the unilateral turning bias of rats when they are confronted with two boards angled at 30°. A neurologically healthy rat will turn to each side equally. In contrast, rats with unilateral neurological deficits are more prone to turn to the non-impaired side because of contralateral vibrissae sensation deficits and rearing motor dysfunction. This asymmetric test was repeated 15 times during each session of evaluation.

### 2.6. Histological Staining and Prussian Blue Staining

At the conclusion of the study, the rats were humanely euthanized, and their brains were rapidly collected, perfusion-fixed, paraffin-embedded, and sliced at a precision thickness of 5 μm. These brain slices were initially stained with eosin and hematoxylin and observed via light microscopy to evaluate general cytoarchitecture. To conclusively identify the presence and spatial distribution of intracellular iron, the brain slices underwent serial rehydration and were then stained with Prussian blue (Millipore-Sigma, St. Louis, MI, USA). The cell nuclei were subsequently counterstained by Fast Red (Millipore-Sigma, St. Louis, MI, USA) to provide morphological contrast. These stained brain slices were digitized and observed by high-resolution light microscopy.

### 2.7. Choroid Plexus Isolation

For the fundamental investigation of paracrine interactions between the choroid plexus (CP) and 293T cells, we isolated the endogenous CP from the brains of naïve rats (*n* = 4). The brain was fixed using forceps, and we meticulously dissected the dorsal part to expose the lateral ventricle. At an exact forceps depth of 3.3 mm, the highly vascularized CP was visualized and carefully collected. The harvested CP tissue was subjected to controlled enzymatic digestion using 0.75% collagenase II at 37 °C with mild centrifugation at 20× *g* rpm for 30–60 min. The digested tissue was then centrifuged at 1500× *g* rpm at 4 °C for 10 min and washed three times with sterile PBS. The isolated CP epithelial cells were cultured with a highly specialized DMEM/F12 mixture at a 1:1 proportion. The DMEM/F12 complete medium was composed of 10% FBS, 4 mM L-glutamine, 5 μg/mL insulin, 200 ng/mL hydrocortisone, 20 μM cytosine arabinoside, 100 g/mL antibiotics (penicillin/streptomycin), and 10 ng/mL epidermal growth factor.

### 2.8. Co-Culture of 293T Cells and CP

The complex, bidirectional paracrine interaction of the CP with 293T cells was evaluated using a semi-permeable Transwell co-culture system for cell proliferation, as described previously [16]. We cultured 3 × 10^3^ 293T cells on the 50 μg/mL laminin-coated porous polycarbonate Transwell membrane (Corning-Costar, Cambridge, MA, USA) in the superior chamber. We cultured either 0.031 g of isolated CP cells or blank culture medium in the lower compartment. We then rigorously evaluated the inverse spatial reaction: we cultured 8 × 10^3^ 293T cells in the lower compartment and placed 0.031 g CP on the superior porous membrane. The co-culture system was maintained under optimal physiological conditions for 14 days, subsequently fixed and treated with 10% crystal violet for 30 min. The incorporated crystal violet was then dissolved in dimethyl sulfoxide (DMSO), and we used a highly sensitive Spark 10M spectrophotometer (Tecan, Männedorf, Switzerland) to precisely detect optical light absorbance.

### 2.9. Immunohistochemistry

Brain slices were fixed with 4% paraformaldehyde solution at room temperature for 15 min. The paraffin wax was completely removed by 1× Trilogy buffer (Cell Marque Corporation, Rocklin, CA, USA) for 10 min under a pressurized, heat-induced epitope retrieval environment. The tissue slices were treated with 0.3% H2O2 for 30 min to eliminate endogenous peroxidase activity. Then we washed the tissue slices with PBS and thoroughly blocked them with 2% bovine serum albumin (BSA) for 60 min.

The tissue slices were subsequently incubated with a panel of highly specific primary antibodies, including anti-nestin antibody (1:250; Abcam, Cambridge, MA, USA), rabbit anti-Ki67 monoclonal antibody (1:150; GeneTex, CA, USA), recombinant anti-GFAP antibody (1:250; Abcam, Cambridge, MA, USA), mouse anti-NeuN monoclonal antibody (1:200; Millipore, MA, USA), and anti-CD68, antibody (1:250; Abcam, Cambridge, MA, USA) and anti-Iba-1 antibody (1:200; Abcam, Cambridge, MA, USA). These antibodies were prepared by dilution with PBS containing 2% BSA at the proportions described above. The tissue slices were treated with primary antibodies overnight at 4 °C and were then rigorously washed with PBS. Following this, the tissue slices were incubated with an SS IHC detection kit (BioGenex, San Ramon, CA, USA) containing biotinylated goat anti-mouse secondary antibody conjugated to the enzyme horseradish peroxidase (HRP) for 30 min. Diaminobenzidine (DAB) was then added for another 5 min to catalyze a dark brown precipitate, and the tissue slices were dehydrated via an ascending ethanol gradient method and cleared with xylene for 10 min. These tissue slices were permanently mounted on Permount and visualized using an inverted light microscope (Eclipse TS80i; Nikon, Tokyo, Japan).

### 2.10. Immunofluorescent Staining for Cell Survival and Apoptosis

Double-label immunofluorescent staining was performed on day-36 brain sections to distinguish surviving 293T cells from iron-laden cellular debris. Sections were incubated overnight at 4 °C with rabbit anti-Ku80 monoclonal antibody (1:400; Cell Signaling Technology, Danvers, MA, USA), a human-specific nuclear marker, followed by DyLight 550-conjugated goat anti-rabbit IgG (1:200; Abcam, Cambridge, CA, USA) for 2 h at room temperature. Apoptosis was detected by FITC-conjugated Annexin V (1:200; BioLegend, San Diego, CA, USA) in Annexin V binding buffer (15 min, room temperature), with DAPI nuclear counterstain (1:1000; Sigma-Aldrich, St. Louis, MO, USA). Sequential single-channel imaging eliminated spectral bleed-through.

### 2.11. Statistical Analysis

Continuous longitudinal data (mNSS, corner test, stroke recovery percentage) were analyzed using two-way analysis of variance (ANOVA) with Group and Day as factors, followed by Bonferroni-adjusted Tukey HSD pairwise post hoc comparisons at each observed timepoint to characterize the temporal emergence of group differences. As a complementary analysis, each animal’s longitudinal trajectory was summarized by the time-normalized area under the curve (AUC = ∫Y(t)dt/[t_max − t_min]) and compared between groups by one-way ANOVA followed by Tukey HSD post hoc; this AUC framework provides a per-animal summary of cumulative therapeutic burden that is robust to missing observations at individual timepoints. Normality of group AUC distributions was verified by the Shapiro–Wilk test (all *p* > 0.08). Data are presented as mean ± standard error of the mean unless otherwise noted; statistical significance was set at α = 0.05.

Sample sizes for the four experimental groups were as follows: no-injection ischemic Control (*n* = 10), saline-injected vehicle Control (*n* = 8), rMSC-ION (*n* = 5), and 293T-ION (*n* = 4). The rMSC-ION and 293T-ION arms generated for this feasibility study follow the 3Rs principle (Replacement, Reduction, Refinement), and the sample size is consistent with prior comparative feasibility studies of intracerebral cell therapy [16,21]. Per the pre-specified inclusion criterion described in Section 2.2—which follows STAIR preclinical recommendations [22] and accepted practice for the rat intraluminal MCAO model [23]—six additional rMSC-ION animals with baseline Day-1 infarct volume below 50 mm^3^ were excluded from this analysis to maintain comparable injury severity with the 293T-ION cohort. We acknowledge this remains an exploratory feasibility study, and the present results should be interpreted as preliminary evidence informing the design of an adequately powered confirmatory trial.

All statistical analyses were performed using PSPP (version 2.0.1; GNU Project, Free Software Foundation, Boston, MA, USA).

## 3. Results

### 3.1. Verification of ION Labeling of rMSC and 293T Cells

**Figure 1:**
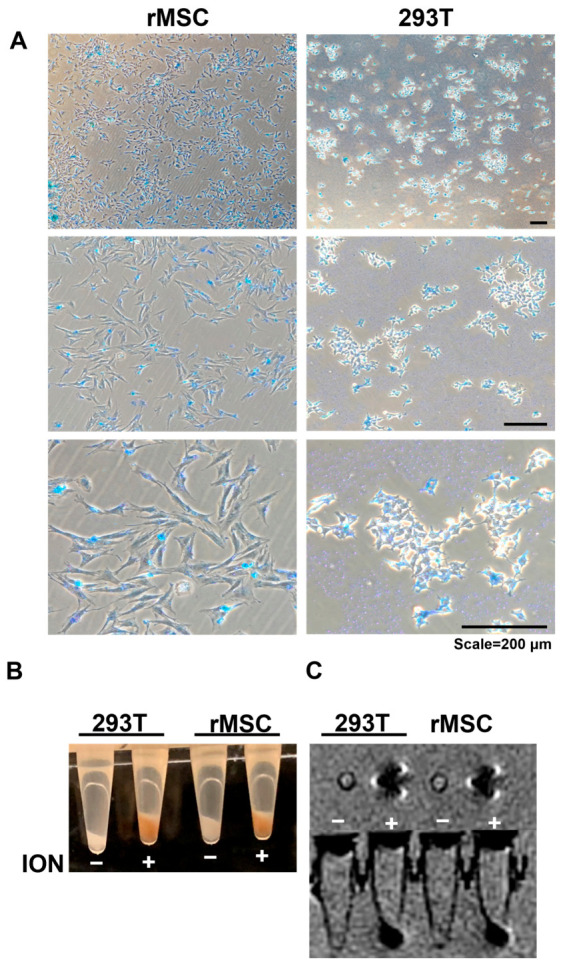
(**A**) Prussian blue staining of rMSC and 293T cells labeled with IONs (293T-ION and rMSC-ION). There is blue staining at the intracellular space of both rMSC and 293T cells, which indicates the presence of loaded iron oxide nanoparticles (IONs). (**B**) Visualization of 293T and rMSC pellets with (+) or without (−) ION labeling. Brownish cell pellets in both the ION-labeled 293T and rMSC were found, whereas cell pellets without ION labeling were white. (**C**) Cellular MRI of cell pellets with (+) or without (−) ION labeling. Both 293T-ION and rMSC-ION cell pellets exhibit dark signals with the same signal intensity in both axial and coronal views.

We conclusively verified the labeling efficiency of rMSC and 293T cells microscopically via Prussian blue staining (Figure 1). The IONs were distinctly visualized as dense blue precipitates sequestered in the intracellular cytoplasmic space of both rMSC and 293T cells. We observed a highly robust ~80% labeling efficiency in both the rMSC and 293T cohorts. High-magnification optical views further demonstrated that the blue ferric staining was localized specifically within the perinuclear endosomal/lysosomal compartments. This was especially prominent in the rMSC group, where the cells exhibited a larger, more spread morphology compared to the compact 293T cells. Importantly, Ferucarbotran labeling at this concentration did not induce cytotoxicity, reactive oxygen species generation, or mitochondrial membrane potential changes in rMSC, and was instead found to enhance rMSC proliferative activity, confirming the biosafety of this protocol [21].

Macroscopically, the ION-labeled rMSC and 293T cells were inspected after being collected as concentrated cell pellets. Both labeled cell pellets exhibited a distinct dark brownish hue, indicating successful massive intracellular ION sequestration, whereas the unlabeled control cell pellets remained pale and translucent. When these cell pellets were subjected to 1.5T clinical MRI, there was a profound, quantitatively identical drop in signal intensity (dark signal voids) under T2-weighted sequences for both the 293T-ION and rMSC-ION cells, confirming their absolute viability as robust contrast agents.

### 3.2. Verification of Stroke Size and Implanted Cells by In Vivo MRI

**Figure 2:**
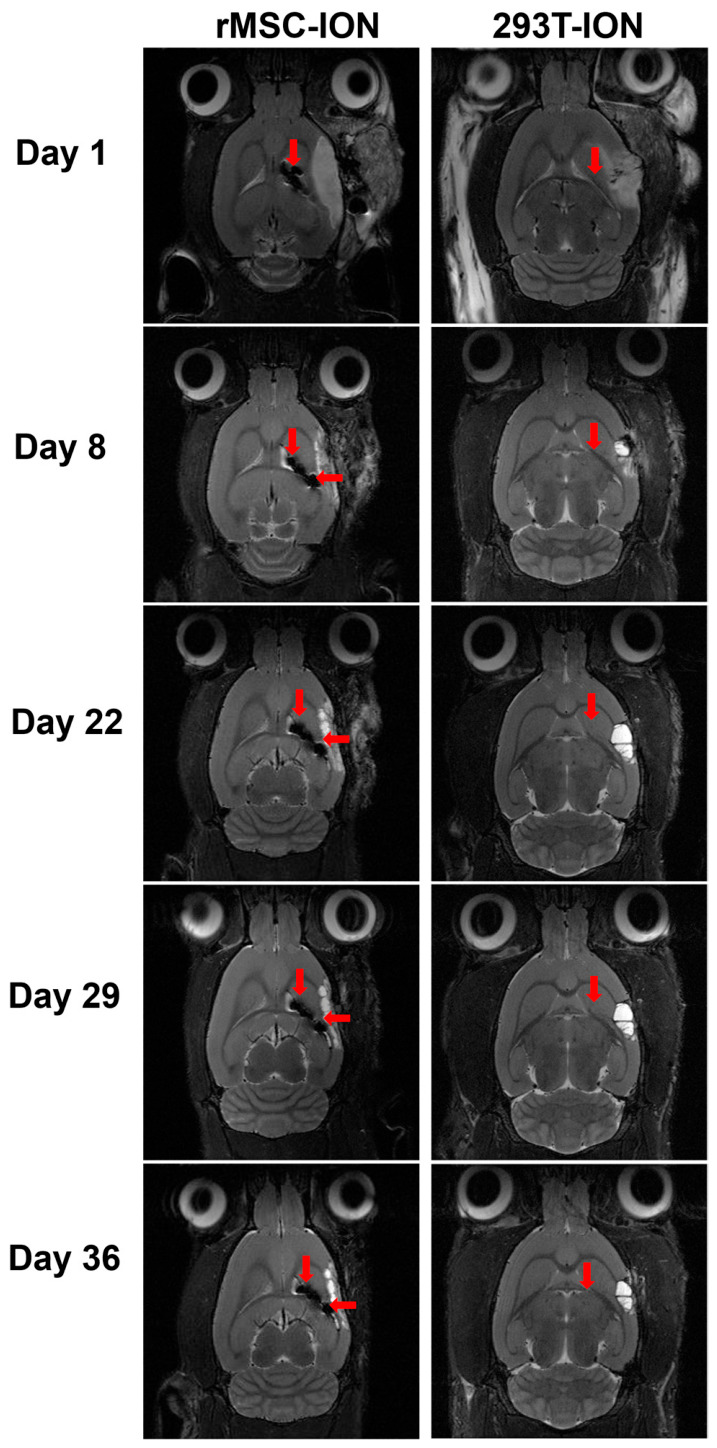
Serial in vivo MR images monitoring ischemic stroke area changes after rMSC-ION and 293 T-ION were injected into the right corpus callosum of ischemic stroke rats. There was a decreased ischemic stroke area in both the 293T-ION and rMSC-ION groups. A low signal intensity area also indicates the initial injected cells in both groups (red arrow). Migratory changes in the low signal intensity area in the rMSC-ION group were observed. However, there was no location change in the low signal intensity across the different time intervals in the 293T-ION group.

The ischemic stroke area and the precise spatial location of the stereotactically implanted cells were longitudinally visualized utilizing ultra-high-field 7T in vivo MRI (Figure 2). Both the 293T-ION and rMSC-ION cohorts demonstrated a visible reduction in the hyperintense stroke lesion size by day 8 post-implantation, which likely reflects the natural resolution of peri-infarct edema along with the initial neuroprotective response.

However, in the temporal serial MRI, we observed a stark divergence in biological behavior. The implanted rMSC-ION exhibited highly targeted migratory behavior, dispersing dynamically from the initial injection site directly toward either the expanding ischemic penumbra or the ipsilateral choroid plexus. Conversely, this active migratory tropism was entirely absent in the 293T group; the dense hypointense signal mass of the 293T-ION remained entirely static and structurally inert at the initial injection coordinates throughout the entire 40-day MR scanning period. The long-term viability of transplanted Ferucarbotran-labeled rMSC in this model is supported by our parallel investigation, which confirmed approximately 28.5% cellular retention with 75% ex vivo viability at day 37 post-transplantation, and further demonstrated via TEM that ION-containing cells maintained an active euchromatic nucleus without evidence of phagocytosed debris [21].

### 3.3. Quantification of Functional Outcomes and Infarct Area Recovery Rate

**Figure 3:**
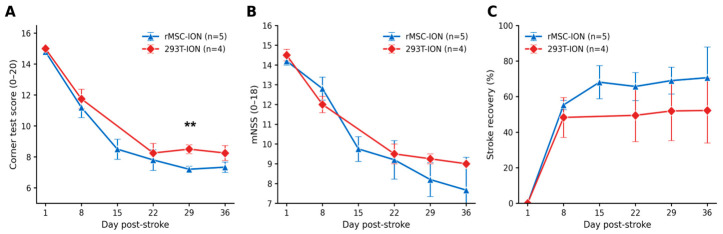
Functional score and recovery rate for infarct size after 293T-ION or rMSC-ION implantation. (**A**) Corner test: rMSC-ION injected rats regained normal test scores on post-implantation day 29, whereas 293T-ION implanted rats fluctuated on the corner test score until post-implantation day 36. (**B**) mNSS score: there was progressive recovery of neurological deficits in both the 293T-ION and rMSC-ION groups. However, neurological recovery was more pronounced in the rMSC-ION group. At the end of the scoring day on post-implantation day 36, the rMSCs-ION group had recovered 2 points better than the 293T-ION group. (**C**) Recovery rate: the recovery rate of both rMSC-ION and 293T-ION cells had a similar pattern. (** *p* < 0.01).

Both 293T-ION and rMSC-ION initiated an early phase of functional rescue, and comprehensive temporal analysis demonstrated that the long-term trajectories of these cell-therapy groups diverged from the second week post-implantation, with rMSC-ION reaching a markedly lower endpoint mNSS than 293T-ION by day 36 (Figure 3). To formally test the cell-therapy effect against vehicle and no-injection baselines, the two-way ANOVA framework incorporated the published no-injection ischemic Control cohort [21] and saline-injected vehicle Control cohort [16] as additional groups (Methods Section 2.2). On the corner test, the four-group analysis revealed a significant Group main effect (F(3,151) = 3.48, *p* = 0.034) with a highly significant Day effect (F(5,151) = 88.74, *p* < 0.001); the cumulative-burden AUC analysis confirmed this group difference (F(3,23) = 3.17, *p* = 0.046), and Tukey HSD post hoc indicated that rMSC-ION animals had significantly lower cumulative sensorimotor deficit than the published ischemic Control animals (*p* = 0.040), whereas 293T-ION animals did not differ significantly from Control (*p* = 0.254). Saline-injected animals did not differ from the no-injection Control (*p* = 0.363), supporting cell-type specificity of the therapeutic effect rather than a nonspecific injection effect. For mNSS, the four-group ANOVA Group main effect was marginal (*p* = 0.068), with rMSC-ION animals trending toward better recovery. For stroke size recovery percentage, the four groups did not differ significantly (*p* = 0.077), consistent with our prior observation [16] that rMSC therapy in this model benefits behavioural recovery without proportional reduction in absolute infarct volume. Figure 3 displays the two cell-therapy intervention groups generated for the present study; the trajectories of the published Control and Saline cohorts can be found in the original publications [16,21].

Both cohorts initially showed progressive recovery as evidenced by a reduction in infarct size. However, the rMSC-ION group demonstrated a consistent, continuous linear recovery trajectory for structural brain size reduction. In sharp contrast, anatomical recovery in the 293T-ION cohort abruptly plateaued; measurable infarct reduction was only observed in the first 8 days (Figure 3A).

In the sensorimotor corner test, the rMSC-ION-injected rats exhibited an accelerated resolution of asymmetric turning biases, successfully regaining normal, stable baseline test scores as early as post-implantation day 29. The 293T-ION implanted rats, however, experienced highly volatile, fluctuating functional scores and were still reported as having statistically abnormal functional deficits at the definitive post-implantation day 36 evaluation (Figure 3B).

Similarly, we observed a progressive decline in absolute neurological deficits in both groups according to the mNSS test; however, the velocity and absolute magnitude of the active functional rescue were significantly more robust in the rMSC-ION group. At the culmination of the scoring on post-implantation day 36, the rMSC-ION cohort significantly outperformed the 293T-ION group by an average functional margin of 2 points (Figure 3C).

### 3.4. Presence, Correlation and Distribution of Microglia and rMSC-ION or 293T-ION

**Figure 4:**
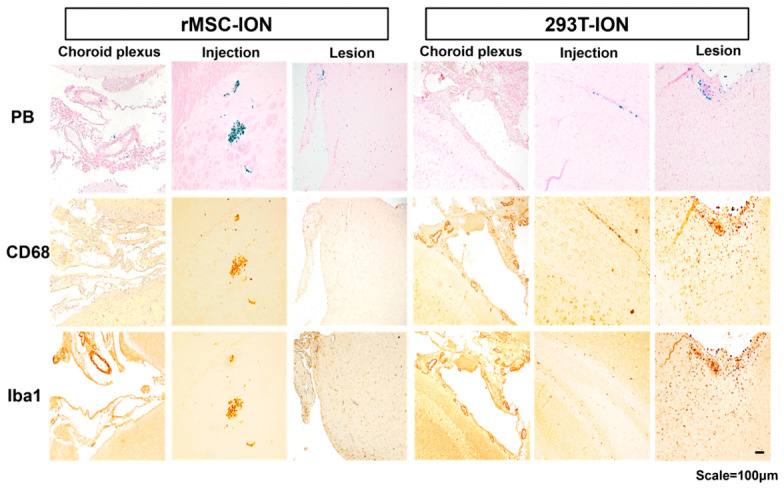
Representative images of Prussian blue and IHC staining of the ischemic–reperfusion injured brain implanted with 293T-ION or rMSC-ION. Prussian blue staining (blue) indicated the presence of the implanted cells, while CD68 and Iba-1 staining(brown) represent microglial activity. Significantly higher microglial activity was observed in the 293T-ION group compared with the rMSC-ION group. The rMSC-ION cells were visualized at the injection site, the choroid plexus (CP), and the lesion site. In contrast, the 293T-ION cells were restricted to the injection site and the lesion site, with no evidence of migration to the CP. (Scale bar = 100 μm).

The anatomical presence and structural integration of the implanted rMSC-ION or 293 T-ION was definitively confirmed by post-mortem Prussian blue histology. To decode the underlying immunological responses, we co-localized activated microglia via CD68 and Iba-1 immunohistochemical (IHC) staining. Microglial activity was profoundly correlated with the spatial physical presence of the implanted cells (Figure 4).

Crucially, there was significantly stronger, highly localized CD68+ and Iba-1+ microglial hyperactivation specifically surrounding the 293T-ION group compared with the rMSC-ION group, indicative of a severe localized pro-inflammatory response. Prussian blue-positive rMSC-ION were systematically identified across multiple regions, including the injection site, the infarct region, and successfully embedded within the epithelial stroma of the choroid plexus (CP). In agreement with the longitudinal MRI signals, 293T-ION remained physically restricted to the injection site and the immediate adjacent lesion boundary. No 293 T-ION was detected in the CP, confirming their lack of long-distance migratory capability.

### 3.5. Qualitative and Quantitative Assessment of Astrocytes, Proliferation Cells, Neuron Differentiation, and Neural Progenitor Cells

**Figure 5:**
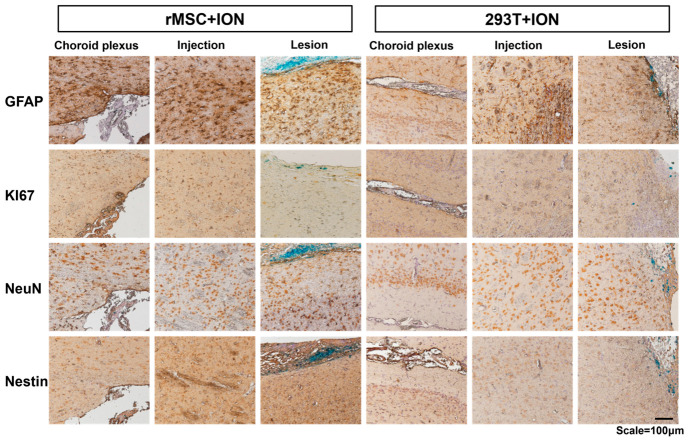
Representative images of rat ischemic–reperfusion brain slices implanted with rMSC-ION or 293T-ION. IHC staining (brown) was performed for GFAP (astrocyte marker), Ki67 (proliferation marker), NeuN (neuronal differentiation marker), and Nestin (neural progenitor marker) in the lesion and choroid plexus (CP). The rMSC-ION group showed a significantly higher number of Ki67+ and Nestin+ cells in both the lesion area and the CP compared to the 293T-ION group. Notably, while rMSC-ION were observed in both regions, the positive signals in the 293T-ION group were primarily localized to the lesion area, with minimal to no expression in the CP. Prussian blue counterstaining identifies the implanted ION-labeled cells (blue) within these regions. (Scale bar = 100 μm).

Through rigorous immunohistochemistry profiling at day 36, we evaluated the induction of reactive astrocytes (GFAP), active proliferative cells (Ki67), mature differentiated neurons (NeuN), and immature neuronal precursor cells (Nestin) within the ischemic lesion boundary and the CP (Figure 5).

**Figure 6:**
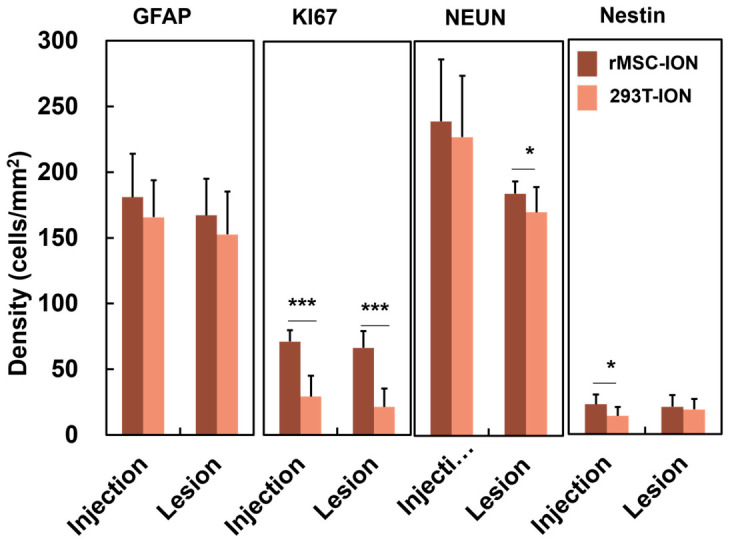
The cell counts were evaluated at the injection site and the ischemic lesion area. IHC analysis revealed that the number of regenerative and progenitor cells following rMSC-ION implantation was significantly higher than that following 293T-ION implantation. Specifically, the rMSC-ION group showed a marked increase in Nestin and Ki67 expression, suggesting enhanced neurogenesis. (GFAP: Glial fibrillary acidic protein; NeuN: Neuronal nuclear protein; Nestin: Neuroepithelial stem cell protein). Data are presented as mean ± SD (* *p* < 0.05, *** *p* < 0.001).

We then quantitatively assessed the absolute cell count density of each neuroplasticity marker at the injection site and within the ischemic lesion. In both regions, the density of remodeling cells, including astrocytes (GFAP+), proliferative cells (Ki67+), mature neurons (NeuN+), and neural progenitor cells (Nestin+), was significantly higher in the rMSC-ION group compared to the 293T-ION group. Notably, there was a highly significant increase in the absolute number of Nestin+ neural progenitors and Ki67+ proliferating cells in the rMSC-implanted cohort compared with the 293T cell group (* *p* < 0.05 to *** *p* < 0.001) (Figure 6). The physical engraftment of rMSC-ION within the CP, and the total absence of 293T-ION in this critical neurogenic niche, were definitively corroborated through these quantitative histological analyses.

### 3.6. Co-Culture of 293T Cells and CP by a Transwell Assay

**Figure 7:**
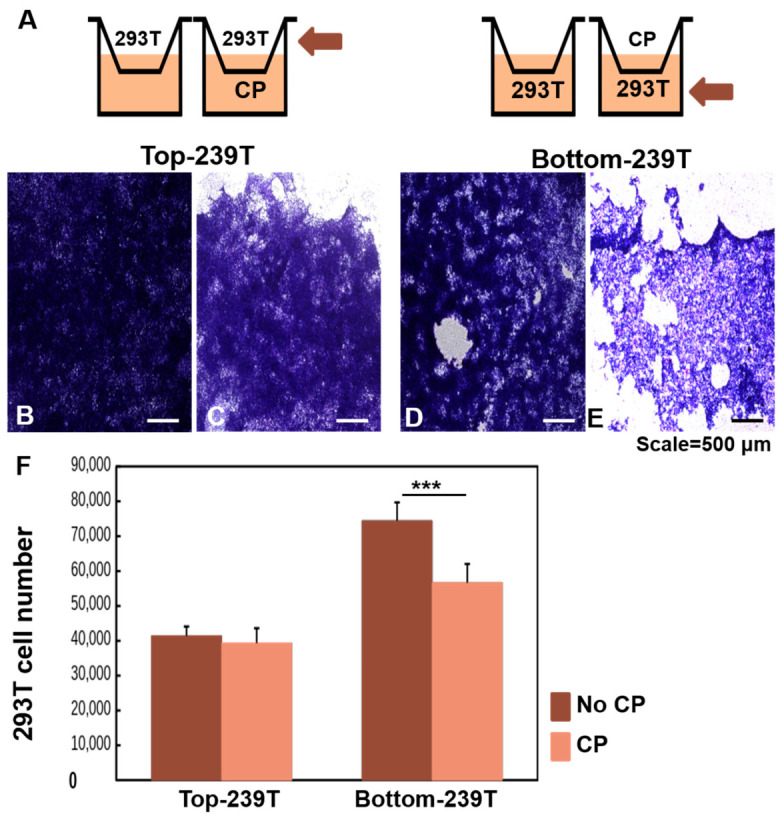
Co-culture of 293T and choroid plexus to evaluate its synergistic proliferation effect by a Transwell assay. (**A**) Schematic of the Transwell system: 293T cells were plated either in the upper chamber with CP in the lower chamber, or vice versa. (**B**,**C**) Methylene blue staining of 293T cells in the upper chamber without (**B**) or with (**C**) CP in the lower chamber. (**D**,**E**) Methylene blue staining of 293T cells in the lower chamber without (**D**) or with (**E**) CP in the upper chamber. (**F**) Quantitative analysis via manual cell counting. A significant suppression effect of CP toward 293T cells was observed when 293T cells were plated in the lower chamber. This inhibitory phenomenon was less significant when 293T cells were plated in the upper chamber. Error bars represent SD (*** *p* < 0.001).

To deconstruct the disparity in in vivo CP tropism, we evaluated the paracrine signaling dynamics between 293T cells and the endogenous CP epithelium using a semi-permeable Transwell assay (Figure 7A).

When 293T cells were plated in the upper chamber, their cellular proliferation remained relatively stable regardless of the presence of CP cells in the lower chamber, as observed by methylene blue staining (Figure 7B,C) and confirmed by manual cell counting (Figure 7F). Under this spatial configuration, the 293T cell densities between the two groups were statistically similar.

However, a distinct inhibitory dynamic was revealed when the spatial configuration was reversed. When 293T cells were plated in the lower chamber, the CP exerted a profound suppression effect. Significantly fewer 293T cells survived and stained at the bottom of the chamber when co-cultured beneath the CP (Figure 7D,E), resulting in a significantly depressed total 293T cell count compared to the control group (* *p* < 0.05, Figure 7F). This suggests that the CP secretome may possess inhibitory factors that negatively influence 293T cell viability or proliferation in close proximity.

### 3.7. Immunofluorescent Demonstration of 293T Cell Apoptosis at the Injection Tract

**Figure 8:**
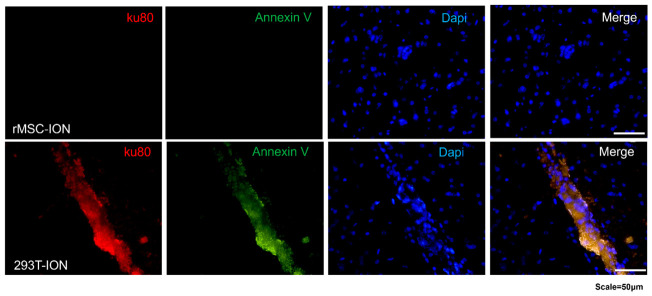
Double-label immunofluorescent demonstration of cell apoptosis at the day-36 injection tract. Ku80 (red, DyLight 550) marks human 293T nuclei; Annexin V (green, FITC) marks phosphatidylserine externalization; DAPI (blue) counterstains all nuclei. The merge (right) shows extensive Ku80^+^/Annexin V^+^ co-positivity confined to the injection tract of the 293T cells but not the rMSC cells. Scale bar = 50 µm.

To distinguish surviving 293T cells from iron-laden cellular debris within the persistent day-36 MRI hypointense signal, brain sections at the injection coordinate were co-stained for the human-specific nuclear marker Ku80 and the early apoptosis marker Annexin V (Figure 8). Ku80-positive nuclei were clearly identified along the injection column in the 293T-ION brain section but not discovered in the rMSC-ION brain section, confirming that human 293T cells remained physically present at day 36. The Annexin V signal extensively coincided with the Ku80-positive cell column, and the merge image showed yellow-orange Ku80^+^/Annexin V^+^ co-positivity spatially confined to the injection tract. These findings indicate that intraparenchymally implanted 293T cells along the injection tract undergo apoptotic cell death rather than persist as viable engrafted cells, providing a cellular correlate for the absence of 293T-ION migration documented by serial 7T MRI and Prussian blue histology (Section 3.2 and Section 3.4).

## 4. Discussion

In the current longitudinal study, we conclusively verified that FDA-approved Ferucarbotran (IONs) are efficiently and spontaneously internalized within the perinuclear endosomal cytoplasm of both rMSC and human neuroendocrine-derived 293T cells. We achieved a robust ~80% labeling efficiency without the requisite use of cytotoxic chemical transfection vectors. Concentrated cell pellets of both lineages confirmed massive intracellular iron sequestration, generating identical, profound T2-weighted signal voids under clinical 1.5T MRI. This validated their absolute viability for high-resolution in vivo spatiotemporal tracking.

The core in vivo findings elucidate a complex biological narrative. While both therapies initially yielded an equivalent day-8 reduction in ischemic stroke volume, their subacute and chronic anatomical and functional recovery trajectories drastically diverged. The rMSC-ION group demonstrated a continuous, sustained recovery, achieving normal sensorimotor baseline scores by day 29. In contrast, the 293T-ION cohort’s recovery abruptly plateaued, yielding poor, fluctuating functional recovery through day 36. Ultra-high-field 7T MRI and Prussian blue histology revealed that rMSC actively migrated toward the ischemic penumbra and specifically integrated into the choroid plexus (CP). The 293T cells, however, remained entirely spatially restricted to the initial injection coordinate. Furthermore, rMSC orchestrated a significantly higher localized density of neuroplasticity markers (GFAP, Ki67, NeuN, and Nestin) compared to the 293T cell therapy.

Several methodological aspects of the present analysis merit emphasis. First, the inclusion of no-injection ischemic Control and saline-injected vehicle Control cohorts—drawn from our previously published studies [16,21] at the same institution under identical surgical and assessment protocols—enables formal statistical testing of the 293T-ION versus rMSC-ION contrast within an integrated four-group framework, the first such analysis combining these cohorts. The corner test analysis demonstrates that rMSC-ION significantly reduces cumulative sensorimotor deficit relative to ischemic Control (Tukey *p* = 0.040), whereas 293T-ION does not (*p* = 0.254), establishing cell-type specificity of the therapeutic effect. Second, the statistical equivalence of saline-injected animals to no-injection Control rules out nonspecific therapeutic effects of intracerebral fluid injection itself, strengthening the interpretation that 293T-ION’s failure to produce functional benefit reflects the cell type’s biological inadequacy rather than a general failure of any intracerebral injection. Third, the absence of a between-group difference in stroke recovery rate (*p* = 0.077 by two-way ANOVA) confirms that rMSC therapy in this model benefits behavioural recovery through paracrine, immunomodulatory, and CP-tropic mechanisms rather than via direct lesion-volume reduction.

The translational relevance of 293T cells requires explicit consideration of their biosafety profile. 293T cells carry the SV40 large T antigen and are immortalized and highly proliferative, properties that preclude direct clinical translation as a whole-cell therapeutic. Within the 40-day observation period, serial 7T MRI did not detect mass-effect lesions, hydrocephalus, or expanding hypointense signal consistent with intracerebral tumor formation; CD68+ clustering remained localized to the injection coordinates without an expanding mass. Definitive long-term tumorigenicity assessment would require extended survival monitoring (≥6 months), histological evaluation for SV40-LT-positive proliferative foci, and cognitive/behavioral assessment for late-emerging effects—none of which were within the scope of this acute feasibility study. We are therefore explicit that 293T cells are not proposed as a directly translatable cellular therapeutic; rather, this study evaluates whether their newly recognized neuroendocrine lineage confers any acute therapeutic activity that could motivate development of either (i) safer non-immortalized derivatives or (ii) acellular fractions (e.g., conditioned medium, exosomes) suitable for translation. We have now directly characterized 293T cell fate at the injection tract using double-label immunofluorescence for the human-specific marker Ku80 and the apoptosis marker Annexin V (Section 3.7, Figure 8); the data indicate apoptotic cell death rather than viable engraftment.

We acknowledge an important interpretive limitation: although CD68 and Iba-1 are reliable markers for activated microglia/macrophages, they do not by themselves distinguish between pro-inflammatory M1-skewed and reparative M2-skewed phenotypes. Our interpretation of an M1-skewed response in the 293T-ION group is therefore inferential, grounded in (i) the persistence and density of CD68+ clustering and (ii) the failure of these animals to enter the chronic neurorestorative phase that depends on M1→M2 transition. Definitive phenotypic characterization with iNOS and CD86 (M1) versus Arg1 and CD206 (M2), multiplexed with Iba-1/CD68 co-staining, is currently in progress in our laboratory using archived FFPE blocks from this study and will be reported in a follow-up communication. Cytokine profiling of perilesional tissue homogenates is similarly planned.

Direct cellular evidence of apoptotic 293T cell fate strengthens the mechanistic interpretation of our findings. The Ku80^+^/Annexin V^+^ co-positivity along the injection tract (Section 3.7, Figure 8) identifies apoptosis as the proximate cellular fate of intraparenchymally implanted 293T cells, resolving whether the persistent day-36 MRI hypointense signal reflects viable cells or iron retained by host phagocytes. This finding is mechanistically congruent with three features of the 293T-ION cohort: absent chemotactic migration to the choroid plexus, since apoptotic cells cannot execute active migration; dense, persistent CD68^+^/Iba-1^+^ microglial clustering, interpretable as a phagocytic response to apoptotic 293T cells and their iron-laden debris; and failure to enter the chronic neurorestorative phase, since apoptotic cells release minimal sustained trophic or paracrine signaling. The temporal ordering of 293T cell apoptosis versus microglial recruitment cannot be determined from a single-time-point analysis and is identified as a follow-up direction.

To the best of our knowledge, our study is the first to directly investigate the un-engineered in vivo intraparenchymal therapeutic behavior of human neuroendocrine-origin 293T cell lines in a severe ischemic stroke rat model. The strategic rationale for investigating 293T cells was driven by recent advanced transcriptomic profiling [9], which definitively demonstrated that HEK293/293T cells possess rich neurofilament expression and cluster with the adrenal medulla and early neural crest lineages, functioning essentially as an immature neuronal archetype [7,8]. While our study revealed that early implantation of 293T cells did facilitate an initial, transient neurological recovery, the chronic anatomical and functional results were vastly inferior to the rMSC cohort.

We observed that the profound recovery mechanisms of rMSC are heavily reliant upon driving endogenous neurogenesis and structural remodeling, evidenced by massive exponential surges in Ki67 (proliferation), Nestin (neural progenitors), and NeuN (mature neurons). While these critical markers were also visualized in the 293T-ION group, their density was sharply attenuated. Beyond basic neuroprotection, a massive mechanistic divergence emerged regarding the host’s innate immune architecture. We documented a profound spatial co-localization of massive, dense clusters of CD68-expressing cells specifically surrounding the static 293T-ION grafts. CD68 is a transmembrane lysosomal glycoprotein serving as the definitive marker for highly phagocytic, activated macrophages and microglial cells [25,26].

Following a severe ischemic insult, microglia execute a highly biphasic, “double-edged” functional role. Necrotic debris triggers resident microglia to rapidly adopt a classically activated, purely pro-inflammatory “M1-like” phenotype, secreting neurotoxic cytokines (e.g., TNF-α, IL-1β) that exacerbate brain gliosis and BBB breakdown [27]. For meaningful structural tissue repair to occur, the microglial population must be therapeutically forced to transition into the alternatively activated “M2-like” repair phenotype, which plays an anti-inflammatory role and secretes critical remodeling factors like IL-10 and insulin-like growth factor (IGF)-1 [28,29]. IGF-1-secreting M2 microglia heavily accumulate in neurogenic niches (SVZ) to drive persistent neurogenesis [30,31]. The intense, sustained CD68+ clustering physically surrounding the 293T-ION heavily implies that the highly proliferative xenograft provokes an aggressive, chronic M1-skewed foreign-body inflammatory response. This persistent local toxicity likely halts any further neurorestoration and explains the plateauing functional recovery of the 293T cohort. Conversely, rMSCs are globally renowned for their profound inherent immunomodulatory prowess, actively evading immune recognition and forcefully driving the neuroprotective M1-to-M2 phenotypic shift via targeted paracrine secretion [32,33,34,35].

The survival of human xenografts in rodent models is a recognized hurdle. Previous studies injecting human leptomeningeal cells into rat brains saw only 1–2.5% survival over two weeks, heavily challenged by massive macrophage infiltration [36]. The viability of transiently implanted 293T cells for molecular imaging has been proven up to 72 h [37,38]. The initial transient recovery observed in our MRI data suggests the 293T cells do release an early, potent burst of neurotrophic factors prior to their immune clearance or immobilization by the M1 microglial attack.

We acknowledge that the comparison of human 293T cells against rat-derived rMSC introduces a species-related immunogenicity confound, and that the elevated CD68+/Iba-1+ microglial response surrounding 293T-ION grafts could, in principle, reflect xenograft rejection rather than an intrinsic biological limitation. Three observations partially mitigate this concern. (i) The persistence of 293T-ION-associated iron deposits and dense CD68+ clustering across the full 40-day observation period substantially exceeds the 1–2.5 week clearance window documented for typical human xenografts in rat brain [36], a temporal pattern more consistent with chronic foreign-body inflammation than with classical immune rejection. (ii) Prior intracerebral implantation of allogeneic non-rMSC cell types in the same model elicits comparatively modest microglial reactivity, suggesting cell-type-specific factors beyond xenogeneity. (iii) The behavioral results show that 293T-ION animals do not differ from no-injection ischemic controls (Tukey *p* = 0.254 on corner test), indicating an absence of therapeutic benefit rather than aggravation. Complete dissociation of immunogenicity from intrinsic cell-biology effects would require parallel testing with a syngeneic transformed cell line as an essential follow-up experiment.

Furthermore, the lack of 293T cell migration was starkly contrasted by the active tropism of the rMSC. We previously described a profound bidirectional cell proliferation enhancement between rMSC and the CP that contributes significantly to infarct size reduction and functional recovery [16]. The CP actively modulates the cerebral spinal fluid (CSF) and is a master regulator of the adjacent SVZ neurogenic niche [39,40,41,42,43,44]. Minimal modifications in the CP secretory profile (e.g., IGFBP-3 and IGFBP-5) can cause profound and lasting neurogenic effects [45]. Our Transwell co-culture assays beautifully deconstructed why 293T cells failed to integrate into this niche in vivo; while heavily diluted, distant CP-derived factors initially promoted 293T proliferation, close physical proximity resulted in the CP exerting a profound suppressive, antagonistic effect on 293T cellular viability.

Additionally, to fundamentally understand why rMSC achieves superior long-term neural network restoration, we must consider electrical integration. The effects of stem cell therapies are traditionally divided into paracrine bystander effects and actual cellular replacement/differentiation [4,46,47,48,49,50]. Crucially, our recent foundational investigations demonstrate that transplanted iron oxide nanoparticle-labeled rMSC do not merely survive anatomically; when magnetically re-isolated ex vivo from the ischemic brains of stroke rats, these Ferucarbotran-labeled rMSC exhibited highly complex, spontaneous neuronal action potential firing on multi-electrode arrays and expressed the mature neuronal marker NeuN [21]. This capacity for true functional electrophysiological differentiation uniquely positions rMSC to rebuild damaged neural circuitry in a manner that unmodified 293T cells apparently cannot sustain.

Our previous investigations consistently demonstrate the robust and versatile therapeutic efficacy of rMSC in ischemic stroke models, firmly establishing their functional superiority over 293T cells [16,21]. We have previously shown that regardless of whether Ferucarbotran-labeled rMSCs are transplanted directly into the ipsilateral hemisphere, delivered to the contralateral lateral ventricle, or stably transduced with red fluorescent protein (RFP), they systematically exhibit active, targeted migration to neurogenic niches like the choroid plexus and yield similar, highly significant trajectories of neurological recovery. This highly reproducible neurorestorative capacity—which is maintained completely independent of the specific intraparenchymal delivery site or the presence of genetic reporter modifications—stands in stark contrast to the spatially static nature and ensuing pro-inflammatory microglial hyperactivation observed with 293T cells. Ultimately, the intrinsic ability of rMSC to overcome spatial delivery variations and consistently drive profound functional recovery further dictates that they remain the vastly preferred cellular vehicle for stroke therapy.

There are some limitations to our study. The exact survival interval of the highly immunogenic human 293T xenograft in the rat brain could not be pinpointed with daily precision. Furthermore, the precise roles of the CD68-expressing cells require further detailed spatial immunofluorescence utilizing definitive M1 (e.g., iNOS) and M2 (e.g., Arg-1) polarization markers to unequivocally map the neuroinflammatory phenotype induced by the 293T cells. Ku80/Annexin V double-label immunofluorescence (Section 3.7, Figure 8) now distinguishes surviving 293T cells from cellular debris and indicates that 293T cells along the injection tract undergo apoptosis. Quantitative ImageJ-based ROI tracking of hypointense signal centroids over time is also being performed to substantiate the qualitative migration findings. The 50 mm^3^ cutoff used as the pre-specified inclusion criterion (Section 2.2) is a laboratory-specific operational definition that should be re-evaluated in any subsequent study using a different surgical protocol or institution. Regarding the absence of a saline control arm, the spontaneous recovery trajectory of this ischemic–reperfusion model following sham intraparenchymal injection has been established in our prior publication using an identical surgical protocol, stereotactic coordinates, and functional assessment battery [16]; repetition of this arm in the current study was therefore not required. Finally, while the xenogeneic nature of 293T cells (human) relative to syngeneic rMSC (rat) represents an inherent design asymmetry, the persistence of 293T-ION-associated iron deposits and inflammatory clusters across the full 40-day observation period substantially exceeds the 1–2.5-week clearance window previously documented for human xenografts in rat brain [36], a temporal pattern more consistent with a chronic foreign-body inflammatory response than with simple immune rejection.

## 5. Conclusions

Both rMSC-ION and 293T-ION cell therapy initiated an acute reduction in infarct size and early functional behavioral recovery. However, rMSC-ION drove a profoundly superior, continuous trajectory of long-term neurorestoration. 293T-ION exhibit a distinctly different mechanistic profile; their lack of chemotactic migration to the choroid plexus and their provocation of massive, localized CD68+ microglial hyperactivation (likely a neurotoxic M1 inflammatory response) ultimately restricts their chronic therapeutic viability as a standalone whole-cell graft. The mechanistic basis for rMSC superiority extends beyond immunomodulation: our group has demonstrated that Ferucarbotran-labeled rMSC magnetically isolated from ischemic rat brains after transplantation exhibit spontaneous neuronal firing activity on multi-electrode arrays and express the mature neuronal marker NeuN, providing direct electrophysiological evidence of functional neural integration that unmodified 293T cells cannot replicate [21]. Nevertheless, 293T cells are instantly available and highly scalable “off-the-shelf” agents, whereas purifying autologous rMSC takes valuable weeks. A highly rational future clinical paradigm may involve the synergistic co-administration of 293T cells (or their purified, cell-free exosomes) to chemically bridge the hyper-acute inflammatory phase, immediately followed by the administration of rMSC to deploy their superior immunomodulatory, CP-homing, and functional electrophysiological integration capacities for long-term stroke recovery.

## Data Availability

The original contributions presented in this study are included in the article. Further inquiries can be directed to the corresponding author.

## Abbreviations

The following abbreviations are used in this manuscript:

MRI	Magnetic resonance imaging
MCAO	Middle Cerebral Artery Occlusion model
rMSC	Bone marrow-derived mesenchymal stem cells
ION	Iron oxide nanoparticles
293T-ION	Iron oxide nanoparticle-labeled 293T cells
rMSC-ION	ION-labeled bone marrow-derived mesenchymal stem cells
DMEM	Dulbecco’s modified Eagle medium
PBS	Phosphate-buffered saline
mNSS	Modified Neurological Severity Score
CP	Choroid plexus
HRP	Horseradish peroxidase
SD	Standard deviation

## References

[1] Campbell B.C.V., De Silva D.A., Macleod M.R., Coutts S.B., Schwamm L.H., Davis S.M., Donnan G.A. (2019). Ischaemic stroke. Nat. Rev. Dis. Primers.

[2] Kawabori M., Shichinohe H., Kuroda S., Houkin K. (2020). Clinical trials of stem cell therapy for cerebral ischemic stroke. Int. J. Mol. Sci..

[3] Jiang Y., Zhu W., Zhu J., Wu L., Xu G., Liu X. (2013). Feasibility of delivering mesenchymal stem cells via catheter to the proximal end of the lesion artery in patients with stroke in the territory of the middle cerebral artery. Cell Transplant..

[4] Kalladka D., Sinden J., Pollock K., Haig C., Mclean J., Smith W., Mcconnachie A., Santosh C., Bath P.M., Dunn L. (2016). Human neural stem cells in patients with chronic ischaemic stroke (PISCES): A phase 1, first-in-man study. Lancet.

[5] Laskowitz D.T., Bennett E.R., Durham R.J., Volpi J.J., Wiese J.R., Frankel M., Shpall E., Wilson J.M., Troy J., Kurtzberg J. (2018). Allogeneic umbilical cord blood infusion for adults with ischemic stroke: Clinical outcomes from a phase I safety study. Stem Cells Transl. Med..

[6] Levy M.L., Crawford J.R., Dib N., Verkh L., Tankovich N., Cramer S.C. (2019). Phase I/II study of safety and preliminary efficacy of intravenous allogeneic mesenchymal stem cells in chronic stroke. Stroke.

[7] Shaw G., Morse S., Ararat M., Graham F.L. (2002). Preferential transformation of human neuronal cells by human adenoviruses and the origin of HEK 293 cells. FASEB J..

[8] Graham F.L., Smiley J., Russell W.C., Nairn R. (1977). Characteristics of a human cell line transformed by DNA from human adenovirus type 5. J. Gen. Virol..

[9] Lin Y.-C., Boone M., Meuris L., Lemmens I., Van Roy N., Soete A., Reumers J., Moisse M., Plaisance S., Drmanac R. (2014). Genome Dynamics of the Human Embryonic Kidney 293 Lineage in Response to Cell Biology Manipulations. Nat. Commun..

[10] DuBridge R.B., Tang P., Hsia H.C., Leong P.M., Miller J.H., Calos M.P. (1987). Analysis of Mutation in Human Cells by Using an Epstein-Barr Virus Shuttle System. Mol. Cell. Biol..

[11] Rio D.C., Clark S.G., Tjian R. (1985). A mammalian host-vector system that regulates expression and amplification of transfected genes by temperature induction. Science.

[12] Debeb B.G., Zhang X., Krishnamurthy S., Gao H., Cohen E., Li L., Rodriguez A.A., Landis M.D., Lucci A., Ueno N.T. (2010). Characterizing cancer cells with cancer stem cell-like features in 293T human embryonic kidney cells. Mol. Cancer.

[13] Sena-Esteves M., Gao G. (2018). Production of high-titer retrovirus and lentivirus vectors. Cold Spring Harb. Protoc..

[14] Chien L.-Y., Hsiao J.-K., Hsu S.-C., Yao M., Lu C.-W., Liu H.-M., Chen Y.-C., Yang C.-S., Huang D.-M. (2011). In vivo magnetic resonance imaging of cell tropism, trafficking mechanism, and therapeutic impact of human mesenchymal stem cells in a murine glioma model. Biomaterials.

[15] Hsiao J.-K., Tai M.-F., Chu H.-H., Chen S.-T., Li H., Lai D.-M., Hsieh S.-T., Wang J.-L., Liu H.-M. (2007). Magnetic nanoparticle labeling of mesenchymal stem cells without transfection agent: Cellular behavior and capability of detection with clinical 1.5 T magnetic resonance at the single cell level. Magn. Reson. Med..

[16] Wu M.-R., Lee C.-H., Hsiao J.-K. (2020). Bidirectional enhancement of cell proliferation between iron oxide nanoparticle-labeled mesenchymal stem cells and choroid plexus in a cell-based therapy model of ischemic stroke. Int. J. Nanomed..

[17] Alphandéry E. (2019). Biodistribution and targeting properties of iron oxide nanoparticles for treatments of cancer and iron anemia disease. Nanotoxicology.

[18] Lu C.-W., Hsiao J.-K., Liu H.-M., Wu C.-H. (2017). Characterization of an iron oxide nanoparticle labelling and MRI-based protocol for inducing human mesenchymal stem cells into neural-like cells. Sci. Rep..

[19] Yu F., Huang T., Ran Y., Li D., Ye L., Tian G., Xi J., Liu Z. (2021). New insights into the roles of microglial regulation in brain plasticity-dependent stroke recovery. Front. Cell Neurosci..

[20] Xiong X.Y., Liu L., Yang Q.W. (2016). Functions and mechanisms of microglia/macrophages in neuroinflammation and neurogenesis after stroke. Prog. Neurobiol..

[21] Huang D.-M., Lu C.-W., Hsiao J.-K. (2025). Transplanted iron oxide nanoparticle-labeled mesenchymal stem cells exhibit ex vivo neuronal firing activity in ischemic stroke rats. Int. J. Nanomed..

[22] Fisher M., Feuerstein G., Howells D.W., Hurn P.D., Kent T.A., Savitz S.I., Lo E.H., STAIR Group (2009). Update of the stroke therapy academic industry roundtable preclinical recommendations. Stroke.

[23] Fernandez-Susavila H., Iglesias-Rey R., Dopico-Lopez A., Perez-Mato M., Sobrino T., Castillo J., Campos F. (2017). Inclusion criteria update for the rat intraluminal ischaemic model for preclinical studies. Dis. Models Mech..

[24] (1999). Stroke Therapy Academic Industry Roundtable (STAIR). Recommendations for standards regarding preclinical neuroprotective and restorative drug development. Stroke.

[25] Chistiakov D.A., Killingsworth M.C., Myasoedova V.A., Orekhov A.N., Bobryshev Y.V. (2017). CD68/macrosialin: Not Just a Histochemical Marker. Lab. Investig..

[26] Jurga A.M., Paleczna M., Kuter K.Z. (2020). Overview of general and discriminating markers of differential microglia phenotypes. Front. Cell Neurosci..

[27] Yenari M.A., Kauppinen T.M., Swanson R.A. (2010). Microglial activation in stroke: Therapeutic targets. Neurotherapeutics.

[28] Qin C., Zhou L.-Q., Ma X.-T., Hu Z.-W., Yang S., Chen M., Bosco D.B., Wu L.-J., Tian D.-S. (2019). Dual functions of microglia in ischemic stroke. Neurosci. Bull..

[29] Ponomarev E.D., Veremeyko T., Weiner H.L. (2013). MicroRNAs are universal regulators of differentiation, activation, and polarization of microglia and macrophages in normal and diseased CNS. Glia.

[30] Thored P., Heldmann U., Gomes-Leal W., Gisler R., Darsalia V., Taneera J., Nygren J.M., Jacobsen S.E.W., Ekdahl C.T., Kokaia Z. (2009). Long-term accumulation of microglia with proneurogenic phenotype concomitant with persistent neurogenesis in adult subventricular zone after stroke. Glia.

[31] Dos Santos I.R.C., Dias M.N.C., Gomes-Leal W. (2021). Microglial activation and adult neurogenesis after brain stroke. Neural Regen. Res..

[32] Zhao H., Wan L., Chen Y., Zhang H., Xu Y., Qiu S. (2018). FASL incapacitation alleviates CD4+ T cells-induced brain injury through remodeling of microglia polarization in mouse ischemic stroke. J. Neuroimmunol..

[33] De Luca S.N., Soch A., Sominsky L., Nguyen T.X., Bosakhar A., Spencer S.J. (2020). Glial remodeling enhances short-term memory performance in Wistar rats. J. Neuroinflammation.

[34] Ferro A., Auguste Y.S.S., Cheadle L. (2021). Microglia, cytokines, and neural activity: Unexpected interactions in brain development and function. Front. Immunol..

[35] Silva N.J., Dorman L.C., Vainchtein I.D., Horneck N.C., Molofsky A.V. (2021). In situ and transcriptomic identification of microglia in synapse-rich regions of the developing zebrafish brain. Nat. Commun..

[36] Degiorgio L.A., Bernstein J.J., Blass J.P. (1997). Implantation of cultured human leptomeningeal cells into rat brain. Int. J. Dev. Neurosci..

[37] Massoud T.F., Paulmurugan R., Gambhir S.S. (2004). Molecular imaging of homodimeric protein-protein interactions in living subjects. FASEB J..

[38] Paulmurugan R., Gambhir S.S. (2005). Firefly luciferase enzyme fragment complementation for imaging in cells and living animals. Anal. Chem..

[39] Segal M.B. (2000). The choroid plexuses and the barriers between the blood and the cerebrospinal fluid. Cell Mol. Neurobiol..

[40] Sakka L., Coll G., Chazal J. (2011). Anatomy and physiology of cerebrospinal fluid. Eur. Ann. Otorhinolaryngol. Head. Neck Dis..

[41] Johansson P.A. (2014). The choroid plexuses and their impact on developmental neurogenesis. Front. Neurosci..

[42] Santos C.R.A., Duarte A.C., Quintela T., Tomás J., Albuquerque T., Marques F., Palha J.A., Gonçalves I. (2017). The choroid plexus as a sex hormone target: Functional implications. Front. Neuroendocrinol..

[43] Xiang J., Routhe L.J., Wilkinson D.A., Hua Y., Moos T., Xi G., Keep R.F. (2017). The choroid plexus as a site of damage in hemorrhagic and ischemic stroke and its role in responding to injury. Fluids Barriers CNS.

[44] Bryniarski M.A., Ren T., Rizvi A.R., Snyder A.M., Morris M.E. (2020). Targeting the choroid plexuses for protein drug delivery. Pharmaceutics.

[45] Huang S.L., He X.J., Li Z.F., Yao L., Yuan G.L., Shi W. (2013). Primary culture of choroid plexuses from neonate rats containing progenitor cells capable of differentiation. Balk. Med. J..

[46] Jackson J.S., Golding J.P., Chapon C., Jones W.A., Bhakoo K.K. (2010). Homing of stem cells to sites of inflammatory brain injury after intracerebral and intravenous administration: A longitudinal imaging study. Stem Cell Res. Ther..

[47] Amer M.H., Rose F.R.A.J., Shakesheff K.M., White L.J. (2018). A biomaterials approach to influence stem cell fate in injectable cell-based therapies. Stem Cell Res. Ther..

[48] Grochowski C., Radzikowska E., Maciejewski R. (2018). Neural stem cell therapy-Brief review. Clin. Neurol. Neurosurg..

[49] Sugaya K., Vaidya M. (2018). Stem cell therapies for neurodegenerative diseases. Adv. Exp. Med. Biol..

[50] Ahmadian-Moghadam H., Sadat-Shirazi M.S., Zarrindast M.R. (2020). Therapeutic potential of stem cells for treatment of neurodegenerative diseases. Biotechnol. Lett..

